# A Self-Organizing Model of the Visual Development of Hand-Centred Representations

**DOI:** 10.1371/journal.pone.0066272

**Published:** 2013-06-14

**Authors:** Juan M. Galeazzi, Bedeho M. W. Mender, Mariana Paredes, James M. Tromans, Benjamin D. Evans, Loredana Minini, Simon M. Stringer

**Affiliations:** Department of Experimental Psychology, University of Oxford, Oxford, Oxfordshire, United Kingdom; The University of Plymouth, United Kingdom

## Abstract

We show how hand-centred visual representations could develop in the primate posterior parietal and premotor cortices during visually guided learning in a self-organizing neural network model. The model incorporates *trace learning* in the feed-forward synaptic connections between successive neuronal layers. Trace learning encourages neurons to learn to respond to input images that tend to occur close together in time. We assume that sequences of eye movements are performed around individual scenes containing a fixed hand-object configuration. Trace learning will then encourage individual cells to learn to respond to particular hand-object configurations across different retinal locations. The plausibility of this hypothesis is demonstrated in computer simulations.

## Introduction

Our understanding of the different functions of the posterior parietal cortex (PPC) and its relevant role in visually guided actions has been expanded and extensively explored over the last few decades [1]. As part of the €dorsal stream€, this region receives various sensory inputs (predominantly from early visual cortical areas) and projects its outputs to several premotor and motor areas within the parieto-frontal network [2]. Single-unit recording studies have shown that both sensory and motor related activity is present in this area [3] and it has been suggested that different parts of the PPC, play a key role in the sensorimotor transformations relevant for goal-directed movements. Further studies have shown that there are specialized regions along the PPC for specific actions and processes, generating further functional subdivisions of this region. For example, the lateral intraparietal area (LIP) has been suggested to play a major role in the production of saccades, helping to focus the gaze toward a visual target [4], [5], while on the other hand the parietal reach region (PRR) seems to be more active when reaches are planned [6]. Anatomically, the PRR includes portions of medial intraparietal area (MIP) and area V6A. This region receives direct visual inputs from early visual areas and projects directly to premotor areas, providing one of the most immediate pathways of visual information into the premotor cortex [7].

It has been suggested that sensorimotor transformations along the parieto-frontal circuit may occur in ordered stages where sensory information is initially represented in a reference frame of the receptor (e.g. retinotopic) and later transformed gradually into representations that would ultimately be encoded in the reference frame of the effector [8]. Compatible with this view, it was found that as part of the initial stages of the visuomotor processing for reaching to a visual target, the PRR encoded the reach vectors in eye-centred coordinates [9]. As the recording sites are moved towards cortical area 5 in the superior parietal lobe (SPL), intermediate representations of both eye and hand-centred coordinates were reported, with the proportion of neurons coding the reach vectors in a purely hand-centred reference frame increasing nearer the cortical surface of area 5 [10] and predominantly in area 5d [11]. Hand-centred representations independent of eye positions have been reported as well in cells in the ventral premotor area (PMv) [12], [13]. A variety of cell responses have been reported also in the dorsal premotor area (PMd), including limb-centred cells [14] as well as cells that seem to encode the spatial relation of the eye, the hand and the target [15].

A variety of neural network models have been proposed to reflect the different stages of sensorimotor transformations and explain some of the response properties found in some neurons of the PPC and premotor areas. For example, an artificial neural network model was proposed for a subset of cells in LIP that was able to transform visual input from eye centred coordinates into a head-centred coordinates [16]. They combined visual information of the target in eye-centred coordinates with eye-position signals, developing visual receptive fields gain modulated by eye positions in the hidden layer, as the authors have reported in some of the cells in LIP. Similarly, Chang et al. [17] found cells in the PRR that were gain modulated by eye position and hand position. In the same fashion, they incorporated a hand-position signal to the Zipser’s et al. [16] neural network model, developing gain-modulated cells in the intermediate layer and hand-centred cells in the output layer. However, both these models used a back-propagation algorithm to train the network, which is not considered biologically plausible because the information to change the synaptic weights is not available locally in the presynaptic terminal. Additionally, not only has the nature and role of gain fields in sensorimotor transformations been questioned [18]–[20], but other reports have shown in fact a mixture of cells responses at the different processing stages. For example cells in the PRR, which were reported to be eye-centred and part of the early stages of visuomotor transformations, include already cells with a hand-centred representation [21].

Other computational approaches have suggested a different form of implementing these transformations more flexibly and robustly, with neurons encoding information in a mixture of representations [19], [22]. Despite their computational advantages, we are still lacking fully unsupervised models that explain how the hand-centred visual receptive fields can arise naturally in the different parts of the parieto-frontal circuit in a plausible self-organizing way.

In the present study we show how hand-centred representations could develop during visually guided learning in a well-established neural network model of visual processing in the primate brain, called VisNet [23], [24]. One of the main virtues of VisNet in contrast with the previously mentioned models for reference frame transformations, is that in VisNet learning is completely unsupervised (i.e. there is no external teaching signal to tell the network what the output should be). Therefore, instead of supervised algorithms (e.g. backpropagation), we use a more biologically plausible learning rule where the synaptic weights are updated locally by associative Hebbian-like learning, in this case for example, a *trace learning* rule [25], [26]. The trace learning rule incorporates a memory trace of recent cell firing activity, which has the effect of encouraging cells to learn to respond to input patterns that tend to occur close together in time. Although VisNet was originally used as a model of the ventral visual stream, it has been subsequently applied to simulate visual processes occurring in the dorsal stream [27]. Both ventral and dorsal streams share architectural similarities, each consisting of a hierarchical series of neuronal layers with competition mediated by inhibitory interneurons within each layer.

We explore the hypothesis that trace learning in the feed-forward synaptic connections between successive neuronal layers in the network is able to encourage neurons at the end of the visual pathway to learn to respond to specific locations of a visual target object with respect to the hand by exploiting natural eye movements including fixational eye-movements like drifts and microsaccades. Trace learning drives neurons in the later layers to learn to respond to input images that occur in temporal proximity. We assume that the eyes are continually performing rapid sequences of eye movements, about any visual scene containing a fixed spatial configuration of a hand and object. Trace learning will then encourage individual cells to learn to respond to particular hand-object configurations across the retinal shifts that occur naturally due to rapid sequences of eye movements (e.g. small drifts or microsaccades). The plausibility of this hypothesis is demonstrated in the computer simulations described below.

### Hypothesis

Neurons have been found in multiple areas along the parieto-frontal network, that respond to the location of a visual target in a hand-centred frame of reference, irrespective of where the target is in the retinal frame of reference [10]–[12], [21]. How might visually driven cells in these areas develop their interesting firing properties?

**Figure 1:**
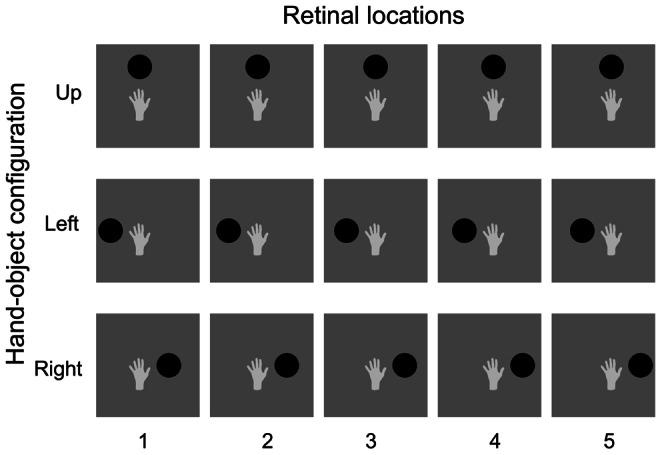
Image sequences. The three image sequences presented to VisNet during training are shown in the separate rows. All of the images in each sequence consist of a hand and a circular visual object. For each image sequence, the visual object is fixed in one of three possible positions with respect to the hand. Top row: visual object is shown in the ‘Up’ location with respect to the hand. Middle row: visual object is shown in the ‘Left’ location with respect to the hand. Bottom row: visual object is shown in the ‘Right’ location with respect to the hand. Each of the three image sequences arises from a series eye movements that result in small shifts in the position of the hand and visual object on the retina (e.g. drifts or microsaccades). The relative positions of the hand and object are unchanged by these microsaccades. During each of the three image sequences, the fixed spatial configuration of the hand and object is translated five pixels at a time towards the right across columns 1 to 5.

The central hypothesis of this paper is that a form of trace learning rule may help neurons in these areas to learn to respond to the location of visual targets in a hand-centred frame of reference in the following manner. During early learning, the visual system is exposed to image sequences similar to those shown in Fig. 1. Each image sequence involves the target object shown in a fixed position with respect to the hand. The eyes are constantly performing movements around the visual scene. Even during fixation, a range of fixational eye movements are performed (e.g. drifts and microsaccades). This has the effect of creating image sequences in which each fixed spatial configuration of the hand and visual object are shifted across the retina. Consequentially, images of a target object in a particular position with respect to the hand, but occurring across different retinal positions, will tend to occur close together in time. In this case, a trace learning rule may be able to associate the images within a particular temporal sequence, corresponding to one particular spatial configuration of the hand and object, with the same subset of output neurons. After enough training, individual output neurons will learn to respond to a particular relative spatial arrangement of the hand and the visual object across all possible retinal locations. This procedure can be repeated for all possible positions of the visual object with respect to the hand. Different output cells should learn to respond to different image sequences corresponding to different positions of the visual object with respect to the hand. We propose that this kind of learning may take place continually as the eyes are moving around the visual environment, even when the subject is not involved in a reaching task.

In the simulations presented below, we demonstrate how trace learning can lead to the development of individual output cells which respond to specific positions of the object with respect to the hand regardless of the retinal locations. We thereby show that trace learning is a potential mechanism for the development of the hand-centred cell firing responses observed in subset of the PPC and premotor areas.

## Methods

### The VisNet Model

**Figure 2:**
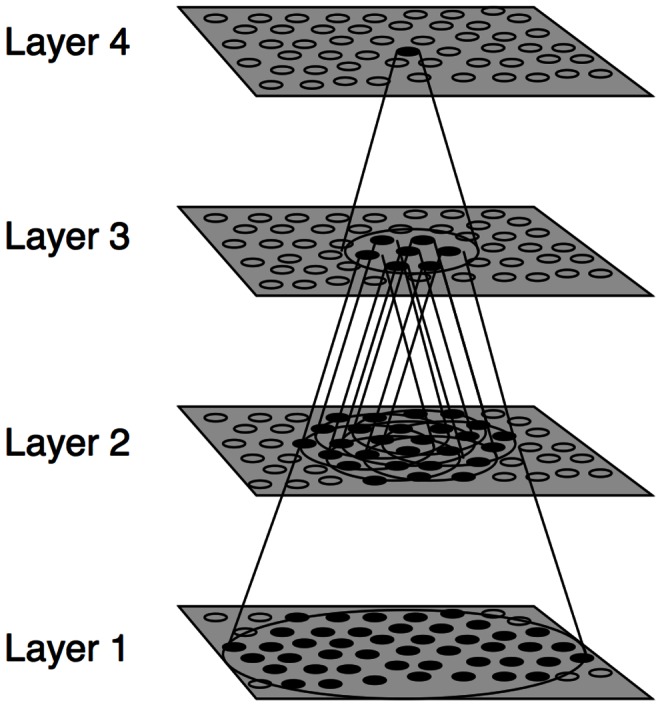
The VisNet model. Stylized image of the VisNet four-layered network. The architecture of the network shows a hierarchical organization which can be found in the dorsal visual system. Convergence through the network is designed to provide fourth-layer neurons with information from across the entire input retina.

The VisNet model consists of a hierarchical series of four feedforward layers of competitive networks. Within each neuronal layer there is lateral competition between neurons implemented by local graded inhibition. During training, there is associative learning at the synaptic connections between the successive layers of neurons (See Figure 2). In VisNet, natural visual images are first passed through an array of filters mimicking the response properties of V1 simple cells, and subsequently these images are fed to the first layer of the network architecture. The forward connections to individual cells are derived from a topologically corresponding region of the preceding layer, using a Gaussian distribution of connection probabilities. These distributions are defined by a radius which will contain approximately 67% of the connections from the preceding layer. This leads to an increase in the receptive field size of neurons through successive layers of the network hierarchy. The network dimensions used for this study are shown in Table 1. The architecture captures the hierarchical organization of competitive neuronal layers that is common in both the dorsal and ventral visual systems.

**Table 1:** Network dimensions.

	Dimensions	Number of Connections	Radius
Layer 4	32×32	100	12
Layer 3	32×32	100	9
Layer 2	32×32	100	6
Layer 1	32×32	100	6
Retina	128×128×32	–	–

Network dimensions showing the number of connections per neuron and the radius in the preceding layer from which 67% are received.

The simulations were conducted utilizing an updated version of the VisNet model [23], [24]. Previous research with VisNet used a difference of two Gaussian function as input filters. In this study, before the stimuli are presented to VisNet’s input layer, they are pre-processed by an initial layer representing V1 with a dimension of 128×128 where each 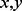-location contains a bank of Gabor filter outputs corresponding to a hypercolumn generated by
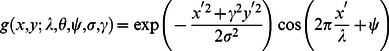
(1)

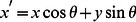
(2)

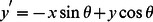
(3)for all combinations of 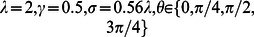 and 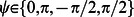.

The activation 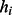 of each neuron 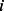 in the network is set equal to a linear sum of the inputs 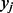 from afferent neurons 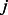 weighted by the synaptic weights 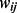. That is,
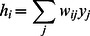
(4)where 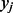 is the firing rate of neuron 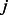, and 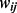 is the strength of the synapse from neuron 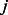 to neuron 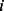.

Within each layer competition is graded rather than winner-take-all, and is implemented in two stages. First, to implement lateral inhibition the activation of neurons within a layer are convolved with a spatial filter, 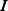, where 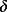 controls the contrast and 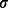 controls the width, and 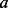 and 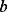 index the distance away from the centre of the filter
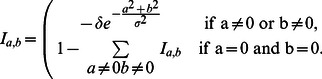
(5)


Typical lateral inhibition parameters are given in Table 2

**Table 2:** Lateral inhibition parameters.

Layer	1	2	3	4
Radius, σ	1.38	2.7	4.0	6.0
Contrast, δ	1.5	1.5	1.6	1.4

Next, contrast enhancement is applied by means of a sigmoid activation function
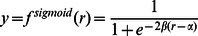
(6)where 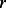 is the activation (or firing rate) after lateral inhibition, 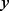 is the firing rate after contrast enhancement, and 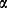 and 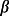 are the sigmoid threshold and slope respectively. The parameters 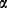 and 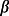 are constant within each layer, although 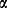 is adjusted to control the sparseness of the firing rates. The sparseness 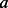 of the firing within a layer can be defined, by extending the binary notion of the proportion of neurons that are firing, as
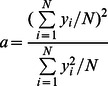
(7)where 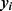 is the firing rate of the 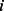
th neuron in the set of 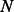 neurons [28], [29]. For the simplified case of neurons with binarised firing rates 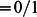, the sparseness is the proportion 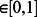 of neurons that are active. For example, to set the sparseness to, say, 5%, the threshold is set to the value of the 95th percentile point of the activations within the layer. Typical parameters for the sigmoid activation function are shown in Table 3.

**Table 3:** Sigmoid parameters.

Layer	1	2	3	4
Percentile	95	95	95	95
Slope 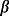	190	40	75	26

The sigmoid parameters used to control the global inhibition within each layer of the model.

For these simulations we used a trace learning rule [25], [26] to adjust the strengths of the feed-forward synaptic connections between the layers during training. The trace rule incorporates a trace 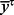 of recent neuronal activity into the postsynaptic term. The trace term reflects the recent activity of the postsynaptic cell. The effect of this is to encourage the postsynaptic cell to learn to respond to input patterns that tend to occur close together in time.

The equation of the original trace learning rule as used by [30] is the following
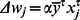
(8)where the trace 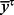 is updated according to
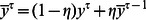
(9)and we have the following definitions


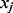
: 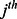 input to the neuron. 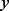: Output from the neuron.


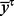
: Trace value of the output of the neuron at time step 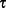. 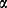: Learning rate. Annealed between unity and zero.


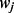
: Synaptic weight between 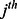 input and the neuron. 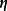: Trace value. The optimal value varies with presentation sequence length.

The parameter 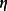 may be set in the interval 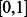. For our simulations the trace learning 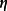 is set to 0.8. If 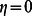 then the equation (8) becomes the standard Hebb rule
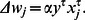
(10)


However, the version of the trace rule used in this paper only includes the trace of activity from the immediately preceding timestep, as used in other studies [24] [31] for improving the performance of the standard trace rule and enhancing the effect of the invariance representation. Thus, the rule takes now the following form
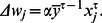
(11)


Neuronal mechanisms that might support trace learning in the brain have been previously discussed [26], [30].

To restrict and limit the growth of each neuron’s synaptic weight vector, 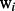 for the 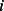
th neuron, its length is normalised at the end of each timestep during training as is usual in competitive learning [32]. Normalisation is required to ensure that the same set of neurons do not always win the competition. Neurophysiological evidence for synaptic weight normalization has been presented [33].

### Stimuli and Training Procedure

The three image sequences presented to VisNet during training are shown in the separate rows of Fig. 1. Each row displays a single sequence consisting of a set of five computer-generated images showing a hand and a circular visual object in a particular spatial configuration. The visual object is fixed in one of three possible positions with respect to the hand (Up, Left and Right). The images belonging to a particular sequence arise from a series of eye movements (e.g. drifts, microsaccades, etc.) and the resulting small shifts in the position of the hand and visual target on the 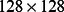 ‘retina’. During each of the three image sequences, the fixed spatial configuration of the hand and object is translated five pixels at a time towards the right across columns 1 to 5.

During the presentation of every image the activation of individual neurons and their firing rates are calculated and subsequently the synaptic weights are updated. The presentation of all three image sequences (i.e. Up, Left and Right) across all five retinal locations constitutes 1 epoch of training. The network is trained one layer at a time starting with layer 1 and finishing with layer 4. In the simulations described here, the numbers of training epochs for layers 1–4 were 50, 50, 50 and 50, respectively.

### Analysis of Network Performance Using Information Measures

Single and multiple cell information theoretic measures are used to assess the network’s performance. Both measures help to determine whether individual cells in the output layer are able to respond to a specific target location in a hand-centred frame of reference over a number of different retinal locations.

In previous VisNet studies, the single cell information measure has been applied to individual cells in the last layer of the network and measures how much information is available from the response of a single cell about which stimulus was shown. In this current study, a stimulus is defined as one of the three different hand-object configurations. If an output neuron responds to just one of the three spatial configurations, and the cell responds to this configuration across all five retinal locations, then the cell will convey maximal single cell information. The amount of information carried by a single cell about a stimulus is computed using the following formula
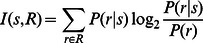
(12)where the stimulus-specific information 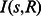 is the amount of information the set of responses 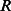 of a single cell has about a specific stimulus (i.e. target location with respect to the hand) 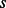, while the set of responses 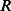 corresponds to the firing rate 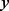 of a cell to each of the three stimuli presented in all five retinal locations. Further details of how the single cell information is calculated are provided in the literature [24], [34].

The maximum single cell information measure is
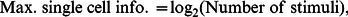
(13)where in this case the number of stimuli, i.e. spatial configurations of the hand and target object, is 3. This gives a maximum single cell information measure of 1.58 bits. This is achieved when the cell responds selectively to just one of the three spatial configurations, and responds to that spatial configuration over all five retinal positions.

On the other hand, the multiple-cell information computes the average amount of information about which stimulus was presented obtained from the responses of all the output cells. This procedure is used to verify whether, across the population of cells, there is information about all of the three stimuli (i.e. hand-object configurations) shown. Procedures for calculating the multiple cell information measure have been described in more detail [24], [35]. In brief, from a single presentation of a stimulus, we calculate the average amount of information obtained from the responses of all the cells regarding which stimulus is shown. This is achieved through a decoding procedure that estimates which stimulus 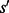 gives rise to the particular firing rate response vector on each trial. A probability table of the real stimuli s and the decoded stimuli 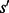 is then constructed. From this probability table, the mutual information is calculated as
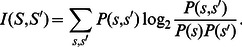
(14)


Multiple cell information values are calculated for the subset of cells which, according to the single cell analysis, have the most information about which stimulus (i.e. hand-object configuration) is shown. In particular, the multiple cell information is calculated from five cells for each stimulus that had the most single cell information about that stimulus. For example, in simulations with three target locations this results in a population of 15 cells. Previous research [36] found this to be a sufficiently large subset to demonstrate that shift invariant representations of each stimulus presented during testing were formed, and that each stimulus could be uniquely identified.

## Results

### Visually Guided Learning of Hand-centred Representations

The purpose of this simulation study was to demonstrate how trace learning can produce cell responses in the output layer of VisNet that are tuned to particular positions of a target object with respect to the hand, irrespective of retinal location. We studied the responses of the output (fourth) layer cells in VisNet before and after the network was trained on the image sequences shown in Fig. 1 as described above.

**Figure 3:**
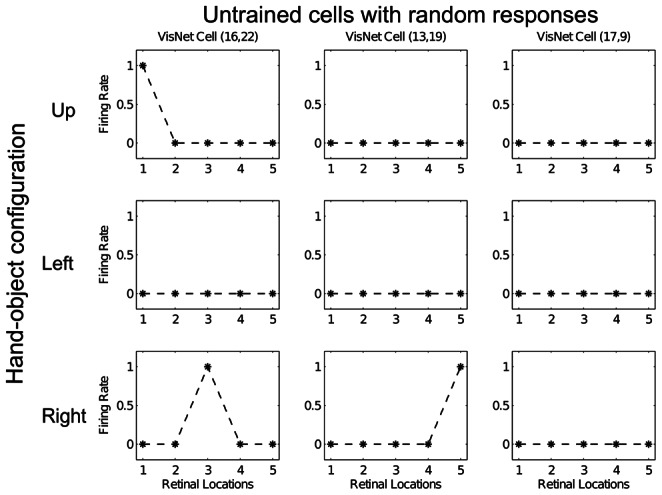
Response profiles of three neurons in the top (fourth) layer of VisNet before training. Each of the three columns shows the firing responses of a particular cell. Each row shows the responses of the cells to one of the three hand-object configurations over all five retinal locations shown along the abscissae. Top row: visual object is shown in the ‘Up’ location with respect to the hand. Middle row: visual object is shown in the ‘Left’ location with respect to the hand. Bottom row: visual object is shown in the ‘Right’ location with respect to the hand. It can be seen that each of the three cells initially responds randomly to each of the hand-object configurations over the different retinal locations.

The response profiles of three neurons in the output layer of VisNet before training are shown in Fig. 3. Each of the three columns shows the firing responses of one particular cell. The three rows show the responses of the cells to the three hand-object configurations across five retinal locations. The top row shows the cell responses when the visual object is in the ‘Up’ location with respect to the hand. The middle row shows the cell responses when the visual object is in the ‘Left’ location with respect to the hand. The bottom row shows the cell responses when the visual object is in the ‘Right’ location with respect to the hand. Fig. 3 shows that, before training, all three cells respond randomly or not at all to the different hand-object configurations.

**Figure 4:**
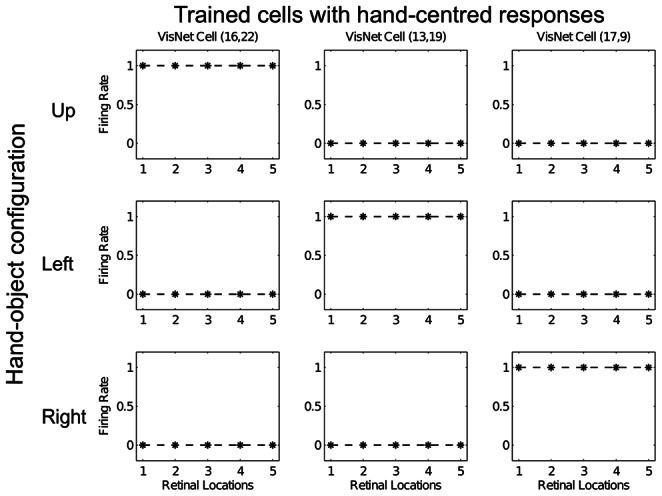
Response profiles of same three neurons in the top (fourth) layer of VisNet after training. Response profiles of the same three neurons from Fig. 3 after training on the images shown in Fig. 1. Conventions as in Fig. 3. It can be seen that each of the three cells responds selectively to just one of the hand-object configurations, and responds to that configuration over all five retinal positions shown along the abscissae. Moreover, each of the three hand-object configurations is represented by one of the cells.

Fig. 4 shows the response profiles of the same three neurons in the output layer of VisNet after training. It can be seen that, after training, each of the three cells has learned to respond to just one of the hand-object configurations, and responds to that configuration over all five retinal locations. The cell in the left column has learned to respond when the visual object is in the ‘Up’ location with respect to the hand. The cell in the middle column has learned to respond when the visual object is in the ‘Left’ location with respect to the hand. The cell in the right column has learned to respond when the visual object is in the ‘Right’ location with respect to the hand. Furthermore, each of the three hand-object configurations is represented by one of the cells.

To provide a more global measure of network performance, we analysed the information carried by the output (fourth) layer neurons in VisNet about which of the three hand-object configurations is presented to the retina. Intuitively, if an output cell has learned to respond perfectly to just one hand-object configuration over all five retinal locations, then it will convey maximal information about which hand-object configuration is currently presented. We, therefore, applied the single and multiple cell information measures described above to the entire population of 1024 neurons in the output layer before and after training.

**Figure 5:**
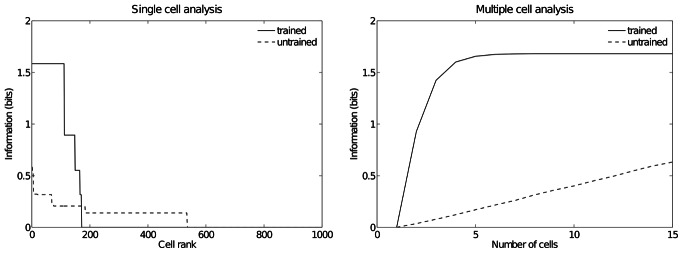
Information analysis. Analysis of the information about where the target object is with respect to the hand conveyed by the output (fourth) layer neurons before and after training. The left plot shows the amount of single cell information carried by individual output cells in rank order. It can be seen that training the network has produced a large increase in the information carried by the output cells. In particular, after training, it was found that 111 cells reached the maximum amount of single cell information of 1.58 bits. In the untrained condition no cells reached maximal information. These cells responded perfectly to just one of the three hand-object configurations, and responded to that configuration across all five retinal locations. The right plot shows the multiple cell information measures calculated across 15 cells with maximal single cell information. It can be seen that, after training, the multiple cell information asymptotes to the maximal value of 1.58 bits. This confirms that all three hand-object configurations are represented by the output cells.

Fig. 5 shows the information measures for the output (fourth) layer neurons before and after training. On the left is shown the single cell information conveyed by individual output cells in rank order. Before training, no cells conveyed the maximal single cell information of 1.58 bits. However, after training, 111 cells had reached this level of single cell information. These cells responded to just one of the three hand-object configurations, and responded to their preferred configuration over all five retinal positions. The right plot shows the multiple cell information measures, which were calculated using 15 cells with maximal single cell information. After training, the multiple cell information is substantially increased and asymptotes to the maximal value of 1.58 bits. The multiple cell information results show that all three spatial configurations of the hand and object are represented by the output cells.

The simulation results described above confirm that trace learning can indeed produce learned neuronal responses which are tuned to a particular location of a visual object in a hand-centred frame of reference, as found in some neurons in the parietal cortex and premotor areas. The key observation is that, after training, the cells respond to specific hand-centred locations regardless of retinal location. The trace learning rule has achieved this by encouraging output cells to learn to respond to images that tend to occur close together in time while the eyes are performing rapid (micro)saccades around the visual scene. Images of a particular configuration of hand and object presented across different retinal positions will tend to occur close together in time. In this case, a trace learning rule can associate all of the images of that spatial configuration with the same subset of output neurons.

### Performance of Model with Different Numbers of Training Epochs

**Figure 6:**
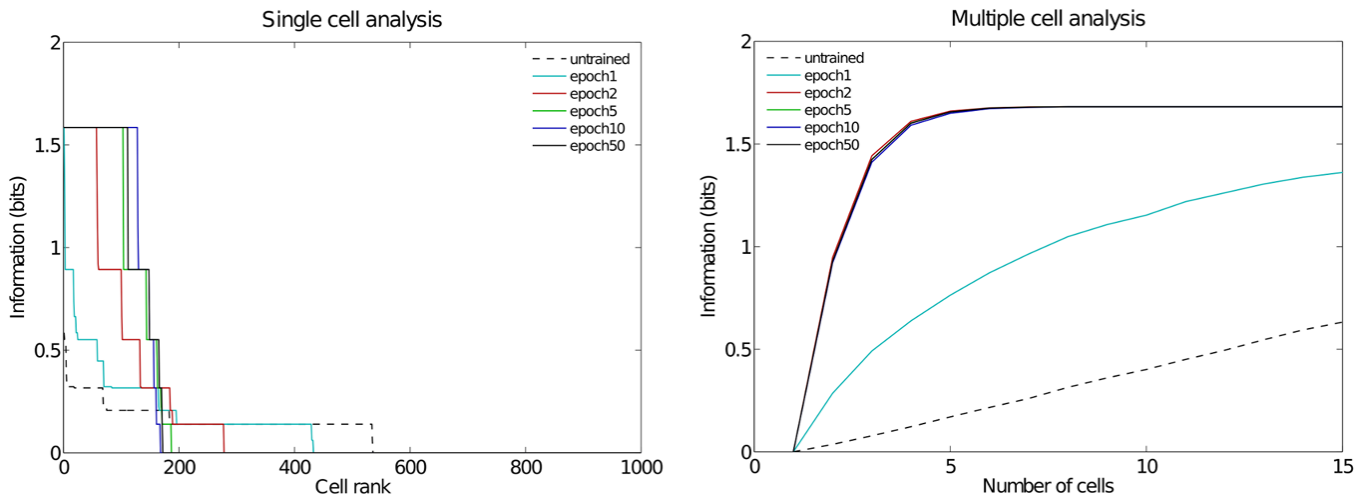
Information analysis for different degrees of training. Analysis of the information about where the target object is with respect to the hand conveyed by the output (fourth) layer neurons for six degrees of training: untrained, 1 epoch, 2 epochs, 5 epochs, 10 epochs, and 50 epochs. The left plot shows the amount of single cell information carried by individual output cells in rank order. After the second epoch it was found that 57 cells reached the maximum amount of single cell information of 1.58 bits. The multiple cell information asymptotes to the maximal value of 1.58 bits after the second epoch. This confirms that all three hand-object configurations are represented by the output cells.

For the previous experiment we also examined the performance of the network as the number of training epochs was reduced. Fig. 6 shows single and multiple cell information analyses for six degrees of training: untrained, 1 epoch, 2 epochs, 5 epochs, 10 epochs, and 50 epochs. The single cell information analysis shows that already after the second epoch of training 57 output cells have achieved the maximum information content. Furthermore, the multiple cell information plot confirms that all of the three spatial configurations are represented by cells which are responding exclusively to one of the spatial configurations and not to any other. These results show that learning of all the spatial configurations occurs quite rapidly (e.g. already after 2 epochs) and remains stable as the number of epochs is increased.

### Performance of the Model as the Density of Training Locations is Increased

**Figure 7:**
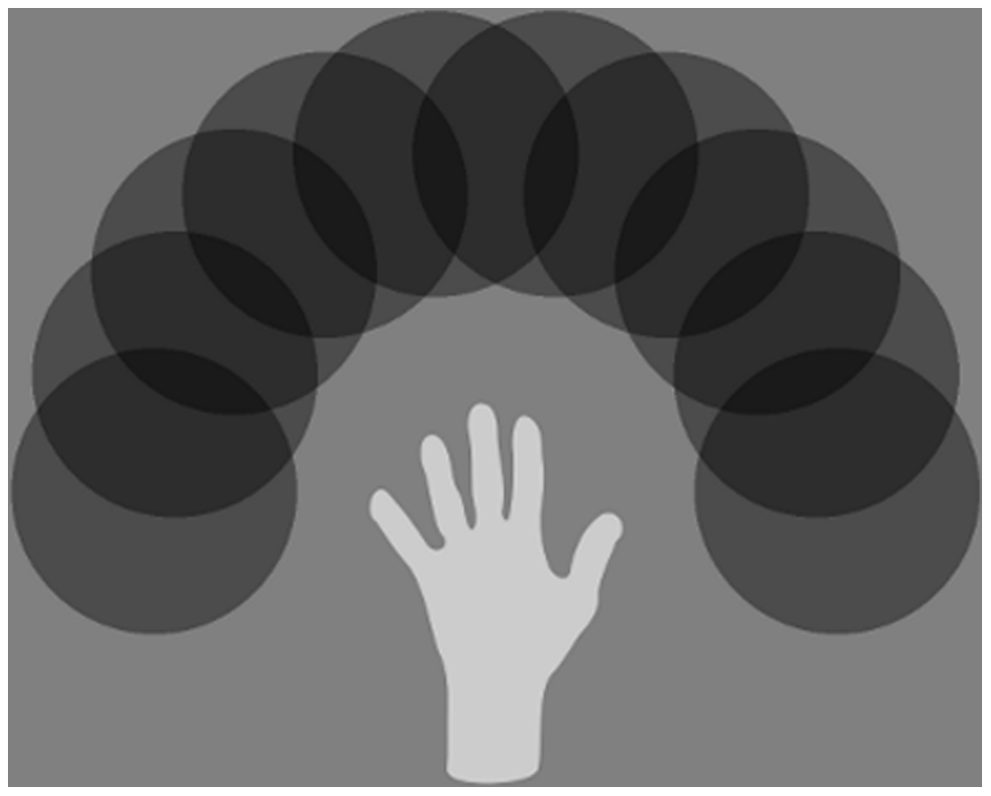
Image of the hand with 10 target locations. With 10 target locations around the hand, the targets are highly overlapping. In this case, the targets begin to form an effective continuum of hand-centred locations.

A key issue is how the network will perform when the number of training locations is increased. In theory, the model should be able to represent a continuum of target locations with respect to the hand. In this section, we explored the performance of the model for eight test cases in which the number of training locations was gradually increased from three to ten in a semicircle around the central hand. The centres of all the objects were evenly distributed along the semicircle (diameter = 36 pixels). The case of ten training locations is shown in Fig. 7. It can be seen that the ten locations effectively form a continuum of locations around the hand.

**Figure 8:**
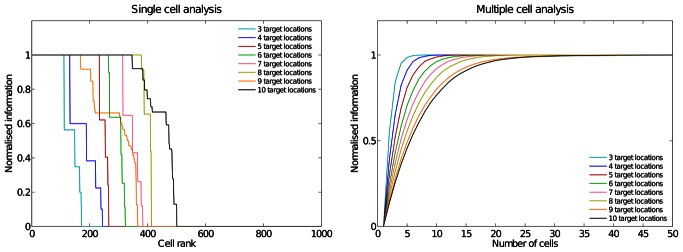
Information analysis as the number of target locations is increased. Analysis of the information conveyed by the output (fourth) layer neurons about the location of the visual target with respect to the hand. Values on the y-axis were normalised by the maximal information possible, 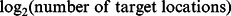, for each simulation. The single cell information analysis (left plot) shows that in all cases more than 10% of the neurons conveyed the maximal single cell information. The multiple cell information measures (right plot) were calculated using five cells for each stimulus that had the most single cell information about that stimulus. For simulations with three target locations this results in a population of 15 cells, while for ten target locations the multiple cell information analysis used 50 cells. It can be seen that in all cases the multiple cell information asymptotes to the (normalised) maximal value, confirming that all of the hand-object configurations are represented by the output cells.

Fig. 8 shows single and multiple cell information analyses for all the test cases. The single cell information analysis shows that in all cases more than a 100 neurons conveyed the maximal single cell information and the multiple cell information plot confirms that in the eight cases, all the configurations are represented by cells which are responding exclusively to one of the spatial configurations and not to any other. In the simulations described here, the numbers of training epochs for all layers were 50, 50, 50 and 50, respectively.

Table 4 summarises for each of the eight test cases the number of cells that are perfectively selective to each one of the target locations with invariance over all five retinal locations. The results are the average of five simulations conducted with identical model parameters, but with different random synaptic weight initializations and different random synaptic connectivities.

**Table 4:** Distribution of responses as the number of spatial locations is increased.

Total number of target locations		Specific target location										
		1	2	3	4	5	6	7	8	9	10	Total
3	Mean	46.0	39.4	36.6	–	–	–	–	–	–	–	122
	SD	3.5	7.9	7.5	–	–	–	–	–	–	–	
4	Mean	44.0	25.6	35.0	30.2	–	–	–	–	–	–	134.8
	SD	8.6	15.7	9.4	13.8	–	–	–	–	–	–	
5	Mean	45.0	43.4	44.4	44.4	41.6	–	–	–	–	–	218.8
	SD	6.3	5.9	6.7	7.8	11.2	–	–	–	–	–	
6	Mean	42.2	32.8	31.0	27.0	40.2	40.4	–	–	–	–	213.6
	SD	10.4	19.2	24.7	18.0	21.5	14.7	–	–	–	–	
7	Mean	44.4	35.4	34.0	28.0	38.2	48.2	47.2	–	–	–	275.4
	SD	10.0	11.3	11.7	19.8	21.6	3.3	4.5	–	–	–	
8	Mean	39.8	42.6	36.2	10.6	10.0	35.4	40.8	47.2	–	–	262.6
	SD	21.3	13.5	15.7	17.8	17.9	22.3	16.1	5.5	–	–	
9	Mean	37.2	31.0	27.6	19.2	10.8	17.0	42.6	32.6	38.0	–	256
	SD	21.1	19.6	15.9	13.1	10.4	18.4	3.5	17.3	19.2	–	
10	Mean	39.8	29.4	36.6	2.8	15.6	6.6	29.0	37.4	46.4	42.6	286.2
	SD	7.4	15.0	15.4	3.0	21.7	9.2	23.7	21.7	4.3	6.9	

This table shows the average number, over five simulations, of perfectly selective neurons responding to each specific spatial configuration of the hand and object across all five retinal locations. Each row corresponds to averaged results and standard deviations from simulations with a fixed total number of target locations. While each column refers to the number of perfect cells found for each specific target location.

We expect individual cells to learn to represent a localised region of hand-centred space as the number of target locations goes to infinity. When the number of target locations was increased to 10, it can be seen from Fig. 7 that the density of locations was approaching an effective continuum. At this point, some cells started to respond to a localised region. For example, in the simulations, when the number of target locations reached 10, there were some cells that had learned to respond to two adjacent hand-centred target locations, in addition to the cells that responded to only one hand-centred target location across all five retinal locations,. If the number of training locations were increased further, we would find that individual neurons learned to respond to a small subset of contiguous locations.

### Performance of the Model with Larger Retinal Shifts

In the simulations described above we used relatively small retinal shifts (i.e. five pixels). However, natural eye movements around any visual scene include larger saccades, which produce greater retinal shifts than the ones we have simulated. Therefore, it is important to test whether the model could learn to respond to a particular hand-object configuration across larger retinal shifts. We hypothesized that since trace learning relies only on the temporal proximity of the input patterns, this learning mechanism should be able to learn to respond to particular hand-object configurations across larger retinal shifts.

In this experiment we explore the performance of the model in the case of larger retinal shifts. However, to do this we needed to increase the size and hence resolution of the retina. In our previous simulations we used a 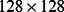 ‘retina’. In order to effectively simulate larger retinal shifts, we doubled the size of the model ‘retina’ (i.e. 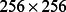
′) and adjusted other network dimensions accordingly. The new dimension values of the network are given in Table 5. All of the other model parameters were the same as in the previous simulations. These dimension changes allowed us to produce larger eye movements that still ensured the hand and object appeared within the visual field.

**Table 5:** Scaled up retina: Network dimensions.

	Dimensions	Number of Connections	Radius
Layer 4	32×32	200	48
Layer 3	32×32	200	36
Layer 2	32×32	200	24
Layer 1	64×64	200	24
Retina	256×256×64	–	–

Network dimensions showing the number of connections per neuron and the radius in the preceding layer from which 67% are received.

We presented the network three image sequences similar to the ones presented initially in Fig. 1. However, during each of the three image sequences, the fixed spatial configuration of the hand and object is translated 35 pixels at a time across the retina instead of 5 pixels.

**Figure 9:**
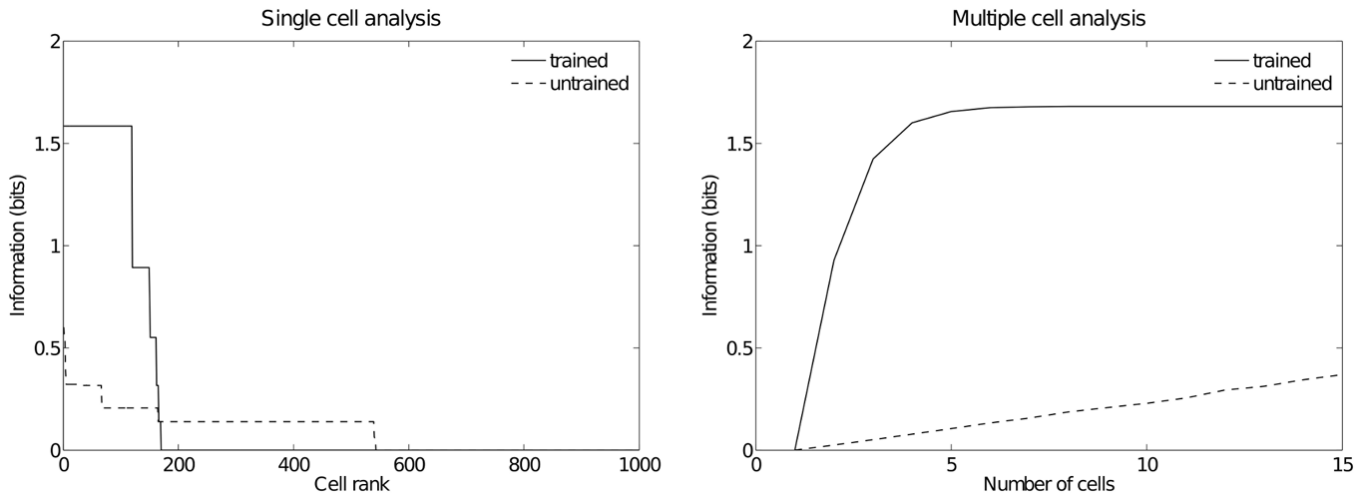
Information analysis for larger shifts. Analysis of the information about where the target object is with respect to the hand conveyed by the output (fourth) layer neurons before and after training. The left plot shows the amount of single cell information carried by individual output cells in rank order. After training, it was found that 119 cells reached the maximum amount of single cell information of 1.58 bits. These cells responded perfectly to just one of the three hand-object configurations, and responded to that configuration across all five retinal locations. The right plot shows the multiple cell information measures calculated across 15 cells with maximal single cell information. It can be seen that, after training, the multiple cell information asymptotes to the maximal value of 1.58 bits. This confirms that all three hand-object configurations are represented by the output cells.

Fig. 9 shows the information measures for the output (fourth) layer neurons before and after training. The single cell information analysis on the left shows that, after training, 119 neurons conveyed the maximal single cell information of 1.58 bits. After training, the multiple cell information is substantially increased and asymptotes to the maximal value of 1.58 bits. This confirms that the three spatial configurations are each represented by cells which are responding exclusively to one of the spatial configurations and not to any other.

In the simulations described here, we can confirm that trace learning can produce learned neuronal responses which are tuned to a particular location of a visual object in a hand-centred frame of reference, even when large eye movements are performed. Images of a particular configuration of hand and object, even if they occur across widely spaced retinal positions, can be associated by trace learning if they occur close together in time.

## Discussion

The results presented in this study have shown how a self-organizing neural network model with a biologically plausible learning mechanism, is capable of generating output cells that are tuned to specific target locations in a hand-centred frame of reference. Furthermore, the response profiles of cells within the network and information measures validate trace learning as the potential mechanism underlying the development of these type of extraretinal representations.

The hand-centred representations in the parieto-frontal circuit are thought to play a role in guiding movement toward visual targets. These neurons, by providing a representation of the location of the target with respect to the hand, are thought to play a role in encoding the vectors for planning a reach towards the visual target. The act of reaching, however, is a complex process that integrates a wide range of sensory and motor information to plan and also control movement trajectories. It is important to emphasize that our model does not pretend to be a model of reaching. The purpose of this study is to provide an account of how cells may self-organize to develop hand-centred representations from eye-centred input, using local learning rules.

### The Role of Visual Signals in the Development of Hand-centred Representations

Our model shows that visual input of the hand and target object could be used to drive the development of hand-centred representations. Similarly, other models present the inputs of the target and the hand visually [22]. More importantly, this assumption is compatible with many experimental findings.

For example, it has been shown in non-human primates that some neurons in PMv with visual receptive fields anchored to the arm, can remap their response to the visible movement of a fake arm instead of the occluded real arm which is stationary [13]. Visual sense of the arm or a realistic fake arm can also generate significant activation in Area 5 and mostly in MIP cells [37], [38]. In humans, hand-centred representations in PPC and premotor cortex have been also remapped to a prosthetic hand during a rubber hand illusion [39].

Additionally, there is also evidence involving visual limb position signals for direct visuomotor transformations between PRR and Area 5 [40]. A portion of V6A neurons have been reported to have an increased activity exclusively for reaching in light conditions and strongly modulated by the sight of the arm [41]. In humans, the superior parieto-occipital sulcus (sPOS) considered as an homologue of the PRR, responds significantly more during direct visual reaching which involves the view of the hand [42]. Moreover, behavioural experiments have shown improvements in the accuracy of reaches when vision of the hand is available, and that vision seems to dominate over other proprioceptive signals [43]–[47].

### The Role of Additional Signals in the Development of Hand-centred Representations

The model presented in this paper shows a biologically plausible learning mechanism (i.e. local learning rule) by which hand-centred visual receptive fields could be developed through visually guided learning. Cells encoding the position of a target in a hand-centred reference frame have been mostly reported in the parietal cortex and premotor areas. However, it is well known that the PPC and premotor areas not only receive bottom-up visual signals [48], [49], but they also receive afferent signals from the somatosensory cortex and top-down motor signals [50], [51]. Experimental studies have shown that, hand-centred cells in PRR and Area 5 can maintain their firing properties when guided only by motor signals or proprioceptive signals in the absence of visual input of the hand and target [10], [52]. Moreover, the delayed-reach paradigm used in many of these studies [10], [53] showed that these neurons continued to maintain their activity when the visual target disappeared from view. This suggests that the hand-centred representations in PRR and Area 5 receive additional proprioceptive input signals specifying the position of the hand, as well as signals conveying a memory of the target location. These additional position signals could be used to update hand-centred representations in the PPC in the absence of visual input.

Positional information of the location of the hand is integrated using inputs from different sensory modalities, including vision and proprioception. Generally, the information from the different sources is congruent helping to make our estimates more precise. Many studies have explored the complex interaction of visual and proprioceptive signals at different stages of motor planning and the generation of reaching movements, and it still remains unclear how and when visual and proprioceptive inputs converge in visuomotor processing [13], [44], [54], [55].

A considerable number of studies have manipulated systematically the availability and congruency between different modalities, affecting and biasing in different ways, the localization of our hands as well as reaching movements. For example, hand position can be significantly biased by mirror-induced illusions, suggesting that visual information is weighted more strongly when the different signals are in conflict [47]. However, other studies have shown the opposite under different paradigms [56].

It is therefore agreed that visual and proprioceptive signals about the location of our hand might have distinct roles and weight differently at different stages of goal-directed movements. When available, visual information of the hand configuration seems to be relevant for the encoding and initial planning of the reach vectors, while proprioceptive signals of the hand position seem to be relevant for transforming a reach plan into the appropriate motor signals [57].

The proprioceptive localization of the hand is generally more precise at distances closer to the shoulder [58]. Localizing the hand using purely proprioceptive signals requires constantly computing and combining the angles of the joints. The representations of the locations of the visual targets with respect to the hand must also be continually updated using the new computed position of the hand. Relying on processing of internal proprioceptive signals will introduce mild error in the estimated hand-centred locations of the targets as well as taking longer to compute. For these reasons, other authors have also suggested that visually derived hand-centred representations, especially in PMv, might be useful for providing more rapid and accurate information of the hand-object configuration for the control of rapid actions [59].

Despite the acknowledged relevance of both vision and proprioception in the planning and execution of motor actions, it has not been established exactly what the actual roles of these signals are in the development of hand-centred visual receptive fields. In this paper we explore a learning mechanism that could be used to generate these extraretinal representations using visual signals representing the location of the object and the hand. The present model assumes that during initial training, the bottom-up visual signals dominate activity in some of these cells. However, hand-centred representations could also be derived from additional proprioceptive cues. Even when it remains unclear how visual and proprioceptive signals converge, the learning mechanism that we have presented here could be implemented using a proprioceptive signal representing the position of the hand. The proprioceptive signals could be either instead of, or in addition to, the visual input of the hand. In future research, we intend to combine the visual network presented in this paper with the proprioceptive representation of the position of the hand, to explain how hand-centred representations in the output layer can be developed by competitive unsupervised training even when the hand is out of sight. In this case, since the proprioceptive signal of the hand position is always present as an input, we would then expect that trace learning could work in a similar way. That is, as the eyes explore a static visual scene, the network could use trace learning to bind together sensory inputs comprised of a combination of the proprioceptive representation of the hand position and a visual representation of a target location. If the proprioceptive signals of hand position entering Area 5 and PMv are particularly dominant, then this may explain why visual representations in these areas are specifically hand-centred rather than in the reference frame of any other object in the visual world. The model presented here provides a novel computational account for how neurons responding in a hand-centred frame of reference might develop in a biologically plausible way by unsupervised visually-guided learning.
